# DLC1 Is a Prognosis-Related Biomarker Correlated With Tumor Microenvironment Remodeling in Endometrial Carcinoma

**DOI:** 10.3389/fonc.2022.823018

**Published:** 2022-02-11

**Authors:** Yalan Wu, Li-e Zheng, Shumin Chen, Chengyu Lv, Yuxiu Huang

**Affiliations:** ^1^ Department of Gynecology, The Affiliated First Hospital of Fujian Medical University, Fuzhou, China; ^2^ Department of Obstetrics and Gynecology, Fujian Maternity and Child Health Hospital, Affiliated Hospital of Fujian Medical University, Fuzhou, China

**Keywords:** deleted in liver cancer 1 (DLC1), endometrial neoplasms, prognosis-related biomarker, tumor microenvironment remodeling, metastasis suppressor gene, macrophages M2, immune cell infiltration

## Abstract

**Background and Aim:**

Deleted in liver cancer 1 (DLC1) is confirmed as a metastasis suppressor gene in endometrial carcinoma (EC). However, its functional mechanisms remain unclear. This study aimed to explore the relationship between DLC1 expression and EC.

**Methods:**

The Cancer Genome Atlas database was used for evaluating the expression of DLC1 in pan-cancer. CIBERSORT was used to assess the relationship between DLC1 and tumor immune infiltration. We applied real-time quantitative polymerase chain reaction to determine the expression of DLC1 in EC and adjacent normal tissue samples. The targeting endogenous protein levels were assessed using the dataset from the cBioPortal database.

**Results:**

DLC1 expression negatively correlated with the clinical characteristics (clinical stage, histologic grade) and positively correlated with the survival of patients with uterine corpus EC (UCEC). The gene set enrichment analysis displayed that the low-expression DLC1 group was enriched in metabolic pathways. Concomitantly, the high-expression DLC1 group was enriched in tumor immune-related activities. The CIBERSORT analysis showed that the number of resting memory CD4 T cells and resting mast cells positively correlated with DLC1 expression, while the number of macrophages M2 had a negative correlation, indicating that DLC1 played a key role in mediating immune cell infiltration. The target gene validation confirmed that DLC1 expression was downregulated in tumor samples. The target protein level was consistently downregulated in tumor samples.

**Conclusions:**

DLC1 levels might be useful in predicting the prognosis of patients with UCEC and especially governing the status of tumor microenvironment transition from immune-dominant to metabolic-dominant. The findings shed a different light on the immune therapeutics of UCEC.

## Introduction

Endometrial carcinoma (EC) is the most common gynecological malignancy with an increasing incidence in developed countries (1, 2). With the development of medicine, a variety of treatments are applied in clinical practice, including surgical resection, chemotherapy, radiotherapy, hormonal therapy, and immunotherapy (3, 4). However, the estimated number of new EC cases in the USA in 2021 is 66,570 with 12,940 deaths according to the American Cancer Society (1). Hence, novel treatment options are urgently needed as the prognosis has not improved over these years.

Many studies have shown that the tumor microenvironment (TME) plays a crucial role in cancer development (5–8). The TME is composed of extracellular matrix, blood or lymphatic vessels, fibroblasts, inflammatory cells, immune cells, and extracellular metabolites. Tumor-infiltrating immune cells (TICs) play a vital role in modulating the TME status of EC (9). Hence, TICs can predict the prognosis of patients with EC. In the TME, tumor cells create a toxic milieu for TICs through metabolism to suppress immunity (10). A study proved that the high-immunity TME might prevent the progression of EC (11). Unfortunately, the concrete mechanism is uncertain. Programmed death-1 signaling was proved to be associated with the activation of tumor-infiltrating CD4 and CD8 T cells in the TME. Based on this, targeting this pathway seems to be a promising immunotherapeutic strategy to be applied in clinical studies (12). A recent study demonstrated a cross talk between tumor cells and tumor-associated macrophages (TAMs) *via* SIGLEC1, CCL8, and CSF1, suggesting that the gene signature of TAMs correlated with poor clinical outcomes (13). These results suggested that the immune response within the TME might be of much importance in the development of EC. Therefore, the dynamic modulation of the immune and stromal components in the TME can be examined through an appropriate precise genetic analysis.

Deleted in liver cancer 1 (DLC1) was initially identified in a primary human hepatocellular carcinoma (HCC) in 1998. It acts as a switch by promoting the conversion of the active RhoGTP into the inactive RhoGDP to affect the cytoskeleton, focal adhesion, and cell migration (14, 15). DLC1 was considered as a metastasis suppressor gene in EC, but its functional mechanisms remain unclear. We speculated that DLC1 was relevant to the prognosis of EC through immune infiltrates. In this study, we conducted a comprehensive assessment of the relationship between DLC1 and patient prognosis using The Cancer Genome Atlas (TCGA) database. We further investigated the link between DLC1 and immune cell infiltration of tumors using CIBERSORT to investigate the aforementioned assumptions. Our results offered novel insights into the functional role of DLC1 in EC, thereby highlighting a potential mechanistic basis whereby DLC1 influenced immune cell interaction with tumors. The restoration of DLC1 expression in cancer cells induced apoptosis and senescence; inhibited cell growth, migration, and invasiveness; and reduced tumor formation (16).

Immunotherapy basically involves stimulating the endogenous immune response specifically against tumor cells and represents the most promising therapeutic approach in EC. DLC1 might be an attractive target for tumor immunotherapy in EC.

## Materials and Methods

### Data Collection

A total of 33 selected cancer types with the corresponding expression profiles and clinical data were downloaded from the TCGA database (https://portal.gdc.cancer.gov/). A dataset of 543 cases of EC was obtained from the TCGA database. We also extracted detailed clinical information, as shown in **Table 1**, including age, histologic grade, histology, clinical stage, diabetes, body mass index (BMI), menopause status, and race. A dataset of a study [Endometrial Cancer (MSK, 2012)] including 95 patients with UCEC (83 endometrioid and 12 serous tumors), with the clinical information and expression profiles, was obtained from the cBioPortal database (http://www.cbioportal.org/). Seventeen pairs of matched EC and adjacent normal tissues were collected from 17 patients with EC who underwent surgery at The Affiliated First Hospital of Fujian Medical University from January 2018 to September 2018. The clinical sample collection cohorts included 17 patients. Reverse transcription-quantitative polymerase chain reaction was carried out to determine the relative expression of DLC1.The patients from the TCGA database were defined as a training cohort, while the dataset from the cBioPortal and the results of PCR were used for external validation.

**Table 1:** TCGA endometrial carcinoma patient characteristics.

Clinical characteristics		Total (543)	%
Age at diagnosis (years)		64 (31–90)	
Histologic grade	G1	98	18.0
	G2	120	22.1
	G3	325	59.9
Histology	Endometrioid adenocarcinoma	401	73.9
	Serous cystadenocarcinoma	137	25.2
	The other	5	0.9
Stage	I	339	62.4
	II	51	9.4
	III	124	22.8
	IV	29	5.4
Diabetes	Yes	99	27.2
	No	265	72.8
BMI	≤30	302	59.0
	>30	210	41.0
Menopause status	Pre	35	7.1
	Peri	445	89.5
	Post	17	3.4
Race	White	372	74.7
	Black or African American	106	21.3
	Asian	20	4.0

### Gene Set Enrichment Analysis

KEGG and C7 gene set v7.4 collections were downloaded from the Molecular Signatures Database as the target sets. Gene set enrichment analysis (GSEA) was performed using the software GSEA-4.1 downloaded from the Broad Institute. We used the GSEA to determine the correlation between DLC1 expression and signaling pathway. The whole transcription of all tumor samples was used for GSEA, and only gene sets supplied with NOM *P* < 0.05 and FDR *q* < 0.07 were considered significant.

### TIC Profile

The CIBERSORT computational method was applied for estimating the TIC abundance profile in all tumor samples, followed by quality filtering. Only 319 tumor samples with *P <*0.05 were selected for the following analysis.

### TIMER Database Analysis

TIMER (https://cistrome.shinyapps.io/timer/) is a database designed for analyzing immune cell infiltrates in multiple types of cancers. We assessed how DLC1 expression correlated with the expression of the markers of particular immune-infiltrating cell subsets.

### Real-Time Quantitative PCR

RT-qPCR was performed using 17 pairs of matched EC and adjacent normal tissues from 17 patients with EC who underwent surgery at The Affiliated First Hospital of Fujian Medical University from January 2018 to September 2018. The study was approved by an institutional review board. All methods were performed following the regulations and relevant guidelines. The written informed consent was obtained before clinical sample collection. The histology of all samples was centrally reviewed by a pathologist. None of the patients had received therapeutic medications or previous surgical interventions before sample collection.

Total RNA from the EC and adjacent normal tissues was extracted using an E.Z.N.A. Total RNA Kit I kit (Omega, Shanghai, China) and then reverse-transcribed into cDNA using a Evo M-MLV reverse transcription reagent kit (Accurate Biology, Hunan, China). The relative expression levels of DLC1 were determined by RT-qPCR using an SYBR Premix Ex Taq kit (TaKaRa, Shanghai, China) in a real-time PCR system. Finally, the threshold cycle (Ct) of the target gene and reference gene was obtained. All samples were set for two replicates, and the final results were averaged. The primer sequences used for amplification are shown in **Table 2**. GAPDH was used as the endogenous controls.

**Table 2:** The primer sequences used for amplification.

Primer name	Primer sequences	Primer length (nt)	Product length (bp)	Annealing temperature (°C)
DLC1 F	AAACAGTATGGCACCTCA	18	164	51.72
DLC1 R	CAATCAAATACCTGGACAA	19		
β-Actin F	TGGCACCCAGCACAATGAA	19	186	60.80
β-Actin R	CTAAGTCATAGTCCGCCTAGAAGCA	25		

### Statistical Analysis

We used R (v.4.0.2) and SPSS 23.0 statistical software to perform statistical analysis. Box plot and violin plot were produced using R language with the ggpubr package. The gene expression difference of DLC1 was analyzed using the Wilcoxon rank-sum test. The survival analysis and clinical characteristic analysis were performed using R language. The survival curve was plotted by the Kaplan–Meier method and log-rank test as the statistical significance test; a *P*-value <0.05 indicated a statistically significant difference. The relationship between clinical characteristics and DLC1 expression level was evaluated using the Wilcoxon rank-sum or Kruskal–Wallis rank-sum test. The cutoff value of DLC1 expression was determined by its median value. We used 2−ΔΔCt to analyze the outcome of PCR. The distribution of paired sample difference disobeying a normality test was found. The median (interquartile range) was used for statistical description of DLC1 expression. The difference of DLC1 expression between matched EC and adjacent normal tissues was analyzed using the Wilcoxon signed-rank test. A *P*-value of less than 0.05 indicated a statistically significant difference.

## Results

### Assessment of DLC1 Expression in Different Cancer and Normal Tissues

We first analyzed the expression of DLC1 in multiple tumor and normal tissue types using the TCGA database to explore DLC1 expression in cancer and normal tissues. The analysis displayed that DLC1 expression significantly declined in normal controls in thyroid carcinoma. In contrast, DLC1 expression significantly decreased in bladder urothelial carcinoma, breast invasive carcinoma, cervical squamous cell carcinoma and endocervical adenocarcinoma (CESC), cholangiocarcinoma (CHOL), kidney chromophobe, kidney renal papillary cell carcinoma (KIRP), liver hepatocellular carcinoma, lung adenocarcinoma, lung squamous cell carcinoma, prostate adenocarcinoma, rectum adenocarcinoma, and uterine corpus EC (UCEC) tissues compared with normal control tissues.

**Figure 1:**
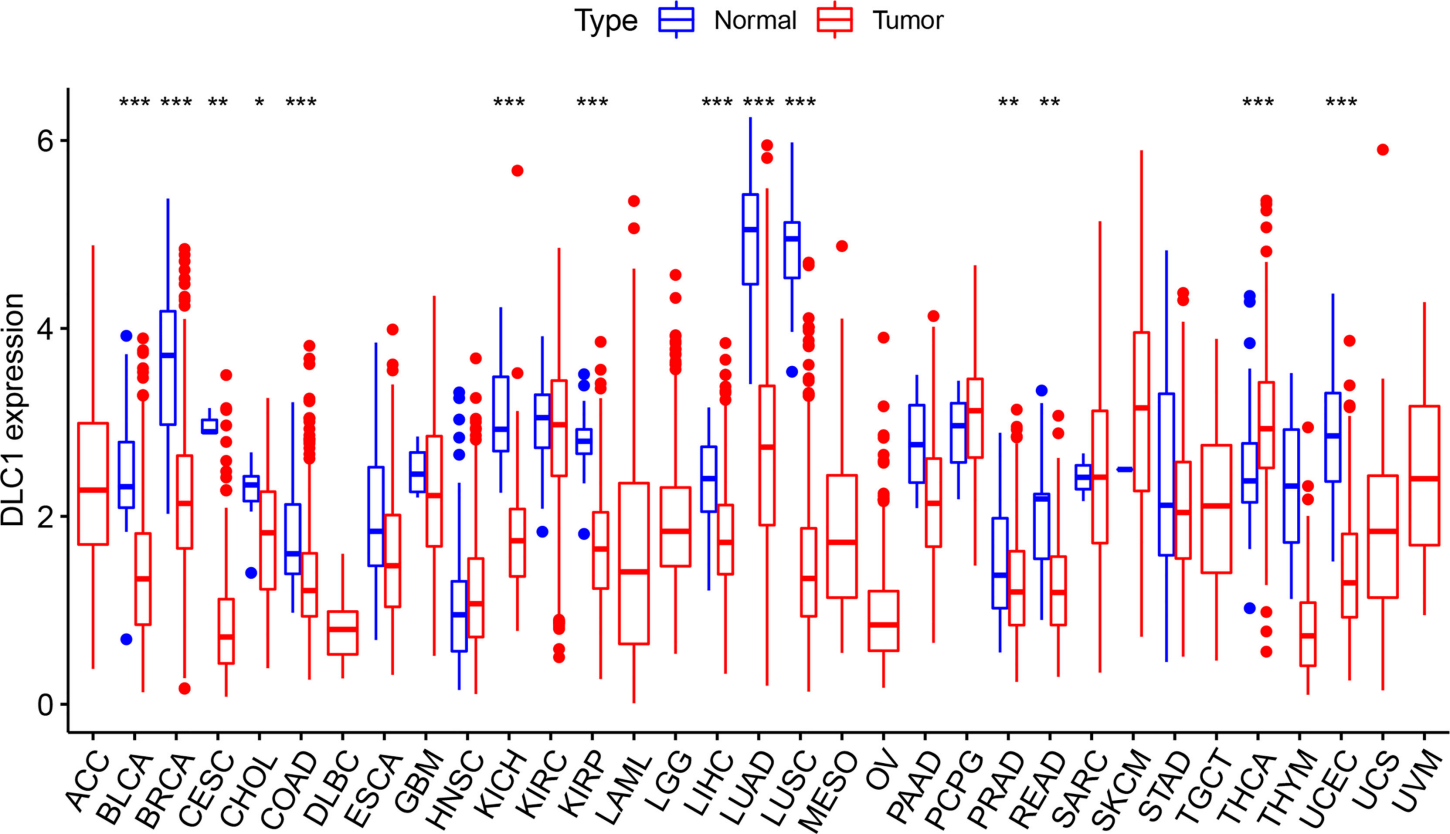
Expression level of deleted in liver cancer 1 (DLC1) in different types of tumor and normal tissues in The Cancer Genome Atlas (TCGA) database (*P < 0.05, **P < 0.01, ***P < 0.001).

The expression of DLC1 in tumor and normal adjacent tissue samples in the TCGA dataset is displayed in **Figure 1**.

### Relationship Between DLC1 Expression and Cancer Patient Prognosis

The study next analyzed the correlation between DLC1 expression and cancer patient prognosis. We employed the TCGA database to assess how DLC1 expression was related to prognosis by analyzing 33 TCGA cancer types. We demonstrated that the increase in DLC1 expression was related to a worse prognosis in ACC [overall survival (OS): *P* = 0.007, hazard ratio (HR) = 1.777, 95% CI = 1.166–2.708; disease-specific survival (DSS): *P* = 0.002, HR = 2.017, 95% CI = 1.306−3.115; progression-free interval (PFI): *P* < 0.001, HR = 2.156, 95% CI = 1.544–3.011] and LGG (OS: *P* < 0.001, HR = 2.431, 95% CI = 1.875–3.152; DSS: *P* < 0.001, HR = 2.765, 95% CI = 2.091−3.655; PFI: *P* < 0.001, HR = 2.178, 95% CI = 1.740−2.725). On the contrary, we found a relationship between decreased DLC1 expression and better patient prognosis in KIRC (OS: *P* < 0.001, HR = 0.682, 95% CI = 0.564–0.826; DSS: *P* < 0.001, HR = 0.618, 95% CI = 0.491−0.777; PFI: *P* = 0.001, HR = 0.738, 95% CI = 0.612−0.890), UVM (OS: *P* < 0.001, HR = 0.287, 95% CI = 0.154–0.537; DSS: *P* < 0.001, HR = 0.259, 95% CI = 0.131−0.513; PFI: *P* = 0.001, HR = 0.417, 95% CI = 0.244−0.713), and UCEC (OS: *P* = 0.002, HR = 0.556, 95% CI = 0.383–0.806; DSS: *P* = 0.006, HR = 0.544, 95% CI = 0.353−0.837; PFI: *P* = 0.007, HR = 0.676, 95% CI = 0.509–0.898) (**Supplement Table 4**).

**Figure 2:**
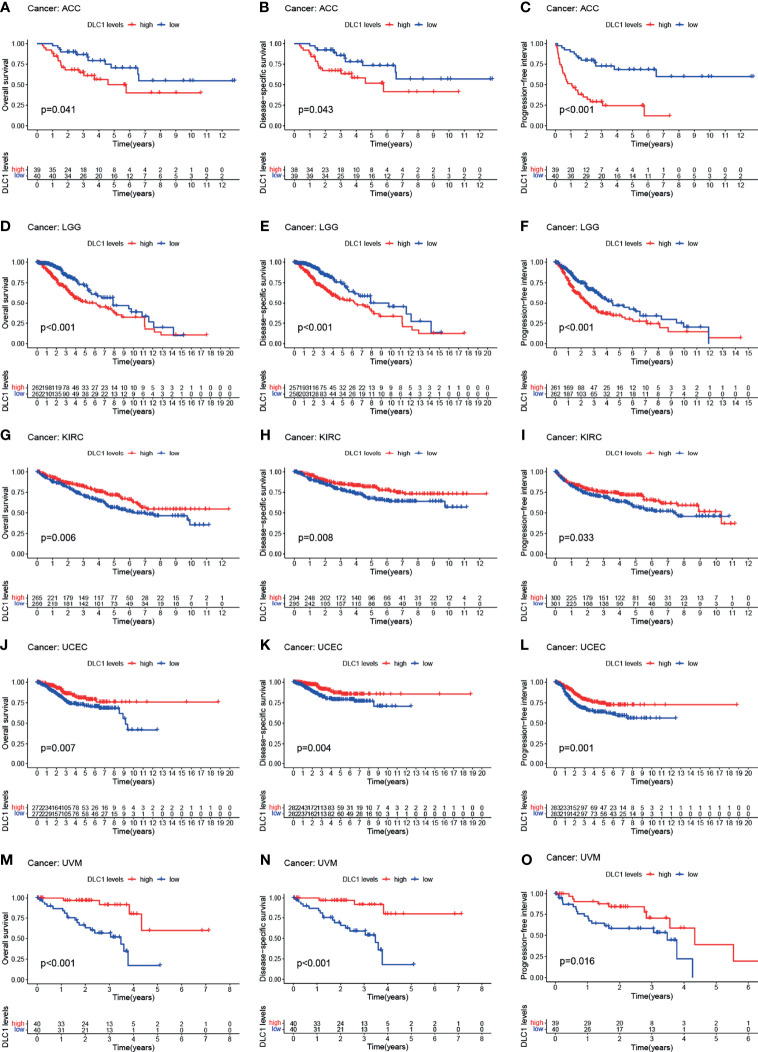
Relationship between DLC1 expression and prognosis of various types of cancer in the TCGA database **(A–O)**. Patients were divided into high-expression and low-expression groups depending on the comparison with the median expression level using the log-rank test. OS, overall survival; DSS, disease-specific survival; PFI, progression-free interval.

**Figure 3:**
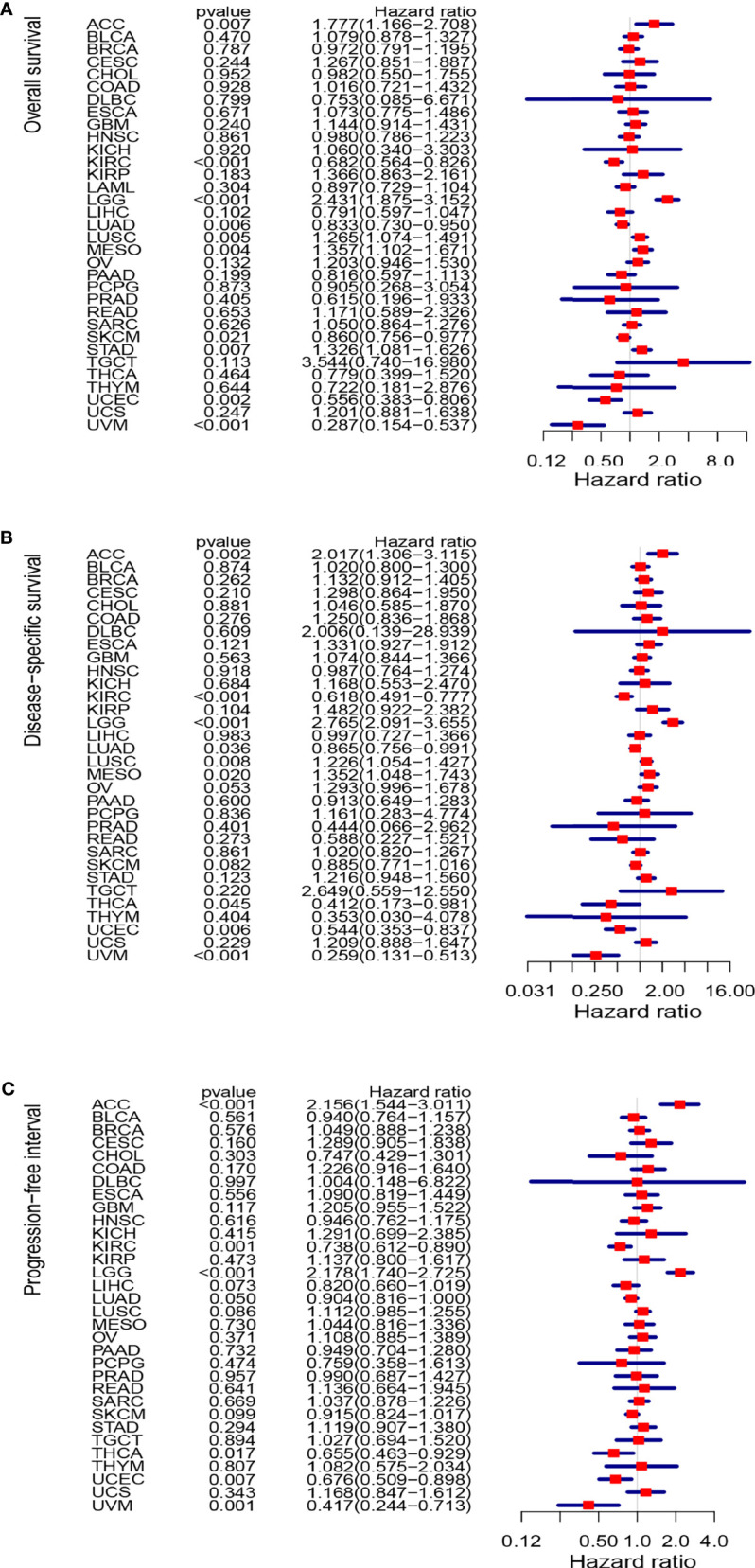
Forest plots of studies investigating the correlation between DLC1 expression and prognosis of various types of cancer in the TCGA database. **(A)** Forest plots for overall survival; **(B)** forest plots for disease-specific survival; **(C)** forest plot for progression-free interval. HR, hazard ratio; CI, confidence interval.

These findings clearly demonstrated that DLC1 expression conspicuously correlated with the outcome of multiple tumor types (**Figures 2**, **3**).

### Decreased Expression of DLC1 in EC Predicts a Poorer Prognosis

We focused on DLC1 expression in UCEC for a subsequent set of analyses, including DLC1 expression and clinical characteristic correlation analysis, survival analysis, and Cox logistic regression analysis.

After excluding normal UCEC samples, DLC1 expression data of 543 patients with EC and clinical data were preserved from the TCGA database (**Table 1**).

**Figure 4:**
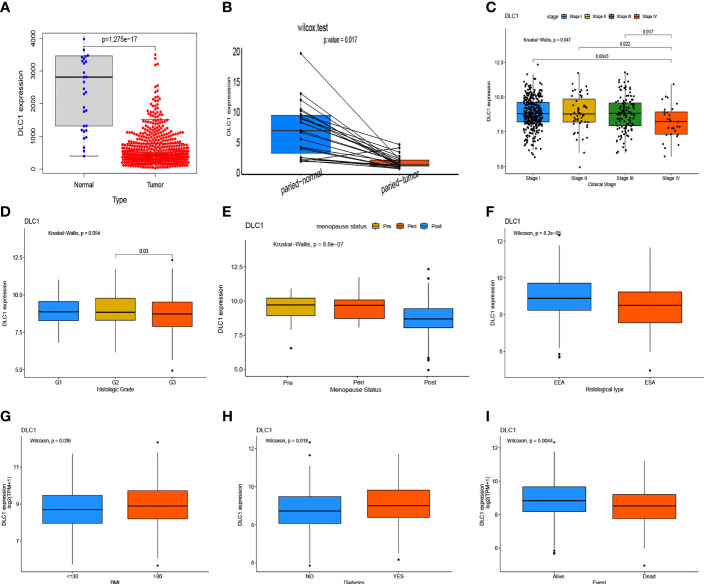
Expression level of DLC1 in samples and its correlation with the clinical characteristics and survival of patients with uterine corpus endometrial carcinoma (UCEC). **(A)** DLC1 expression in the normal and tumor samples. Analyses were performed across all normal and tumor samples with *P*-value close to zero using the Wilcoxon rank-sum test. **(B)** Paired differentiation analysis for the DLC1 expression in the normal and tumor samples derived from the same patient (*P* = 5.572602e−05) using the Wilcoxon signed-rank test. **(C–I)** Relationship between DCL1 expression and clinical characteristics and survival. The statistical significance was calculated using the Kruskal–Wallis rank-sum test or Wilcoxon rank-sum test.

In this study, the Wilcoxon rank-sum test displayed that the DLC1 expression significantly decreased in the tumor samples compared with normal ones (**Figure 4A**). Similar results were observed in the pairing analysis between the normal and tumor tissues that originated from the same patients (**Figure 4B**). Next, using the Wilcoxon rank-sum test and the Kruskal–Wallis test, we discovered that the reduced expression of DLC1 was significantly related to tumor histological types (EEA vs. SEA, *P* = 8.2e−05), histological grade (G2 vs. G3, *P* = 0.03), clinical stage (*P* = 0.047), BMI (*P* = 0.036), diabetes (*P* = 0.018), event (*P* = 0.004), and menopause status (*P* = 8.6e−07) (**Figures 4D–I**). As shown in **Table 3**, age, stage, grade, and DLC1 expression were demonstrated as independent prognostic indicators for patients with UCEC (*P* < 0.05). Multivariate Cox analysis displayed that poor OS had a significant correlation with age (*P* < 0.001, HR = 1.047, 95% CI = 1.019–1.076), stage (*P* < 0.001, HR = 1.859, 95% CI = 0.538–2.325), grade (*P* = 0.001, HR = 2.569, 95% CI = 1.458–4.525), and DLC1 expression (*P* = 0.003, HR = 0.695, 95% CI = 0.547–0.882).

**Table 3:** Univariate analysis and multivariate analysis of the correlation of DLC1 expression with OS among endometrial carcinoma patients.

Parameter	Univariate analysis			Multivariate analysis		
	HR	95% CI	*P*	HR	95% CI	*P*
Age	1.041	1.020–1.062	**<0.001**	1.047	1.019–1.076	**<0.001**
Stage	1.976	1.641–2.381	**<0.001**	1.859	0.538–2.325	**<0.001**
BMI	0.99	0.968–1.013	0.405	1.015	0.987–1.043	0.304
Grade	2.718	1.792–4.123	**<0.001**	2.569	1.458–4.525	**0.001**
Diabetes	1.176	0.685–2.016	0.557	1.118	0.618–2.023	0.713
DLC1	0.827	0.690–0.992	**0.041**	0.695	0.547–0.882	**0.003**

Bold values indicate P <0.05.

HR, hazard ratio; CI, confidence interval.

### DLC1 Might Be Associated With the Immune and Stromal Components in the TME

**Figure 5:**
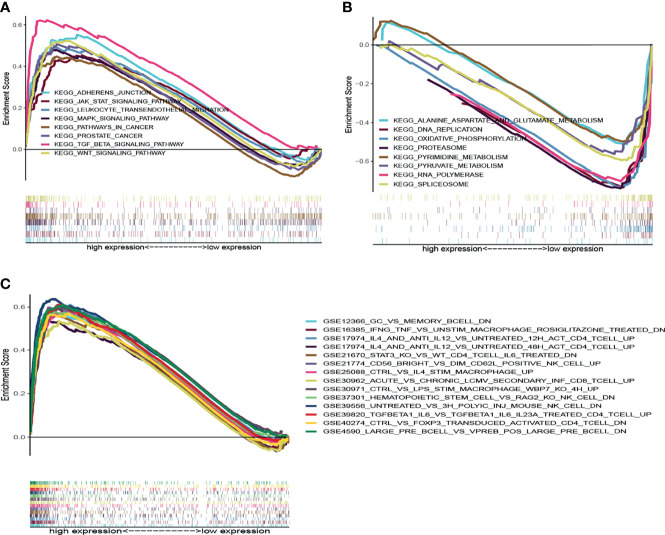
GSEA for samples with high-DLC1 and low-DLC1 expression. **(A)** Enriched gene sets in the KEGG collection of the high-DLC1 expression sample. Each line is uniquely colored demonstrating one particular gene set. Upregulated genes are located on the left approaching the origin of the coordinates, while the downregulated ones lay on the right of the *x*-axis. Only gene sets satisfied with NOM *P <*0.05 and FDR *q <*0.07 were considered significant. In the plot, only leading gene sets are presented. **(B)** Enriched gene sets in the KEGG collection of low-DLC1 expression sample. **(C)** Enriched gene sets in C7 collection and the immunologic gene sets in the high-DLC1 expression sample. Only leading gene sets are displayed in the plot.

Given the DLCI expression was associated with the survival and clinical characteristics of patients with UCEC, GSEA was performed in the high-expression and low-expression groups relative to the median level of DLCI expression. The tumor immunity- and inflammation-related activities, such as Wnt signaling pathway, leukocyte transendothelial migration, and JAK–STAT signaling pathway, were mainly enriched in the high-DLC1 expression group (**Figure 5A**).

In the low-DLC1 expression group, the metabolic pathways that were enriched included pyrimidine metabolism, pyruvate metabolism, alanine aspartate and glutamate metabolism, and oxidative phosphorylation (**Figure 5B**). Regarding the C7 collection defined by MSigDB, multiple immune functional gene sets were predominantly enriched in the high-DLC1 expression group (**Figure 5C**). Nevertheless, no gene sets were enriched in the low-DLC1 expression group. Based on the aforementioned results, we postulated that DLC1 might be associated with the immune and stromal components in the TME.

### Relationship Between DLC1 and the Proportion of TICs

**Figure 6:**
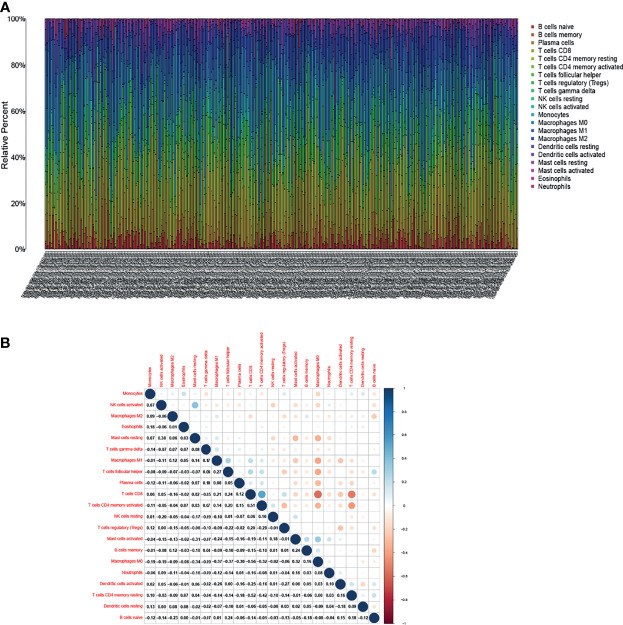
Tumor-infiltrating immune cell (TIC) profile in tumor samples and correlation analysis. **(A)** Proportion of 21 kinds of TICs in UCEC tumor samples is displayed by a ballot plot. Column names of the plot correspond to sample ID. **(B)** Heatmap presenting the relationship between 21 kinds of TICs and numeric in each tiny box representing the association *P*-value between two kinds of cells. The shade of each tiny color box indicates the corresponding association value between two cells. The significance test was displayed using Pearson correlation coefficient.

**Figure 7:**
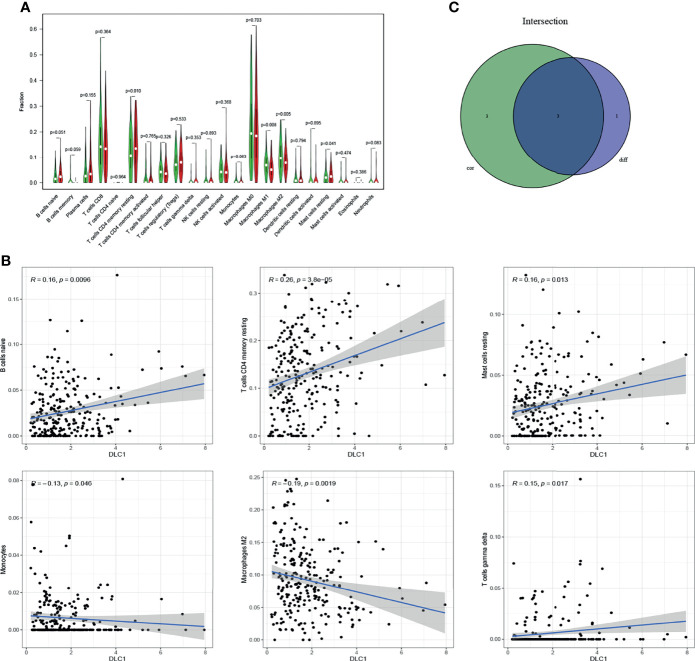
Relationship between TIC proportion and DLC1 expression. **(A)** Differentiation of 21 kinds of immune cells in UCEC tumor samples with low- or high-DLC1 expression compared with the median of DLC1 expression level was shown by a violin plot. The significance test was displayed using Wilcoxon rank-sum. **(B)** Scatter plot showed the relationship between six kinds of TICs in proportion with the DLC1 expression (*P* < 0.05). The blue line in each plot was fitted into a linear model indicating the proportion of tropism of the immune cells along with DLC1 expression; the correlation test was displayed using the Pearson correlation coefficient. **(C)** Venn plot presented three kinds of TICs relative to DLC1 expression co-determined by difference and correlation tests indicated in violin and scatter plots, respectively.

The study further verified the relationship between DLC1 expression and the immune microenvironment. We applied the CIBERSORT algorithm to analyze the proportion of tumor-infiltrating immune subsets and constructed a profile including 21 kinds of immune cells in UCEC samples (**Figure 6**). A total of three kinds of TICs were associated with DLC1 expression, as detected by the difference and correlation analyses (**Figure 7**).

Among these, the proportion of resting memory CD4 T cells and resting mast cells positively correlated with DLC1 expression. However, the number of macrophages M2 had a negative correlation. The results confirmed that the expression levels of DLC1 influenced the immune activity of the TME.

### Target Gene Expression Validation

**Figure 8:**
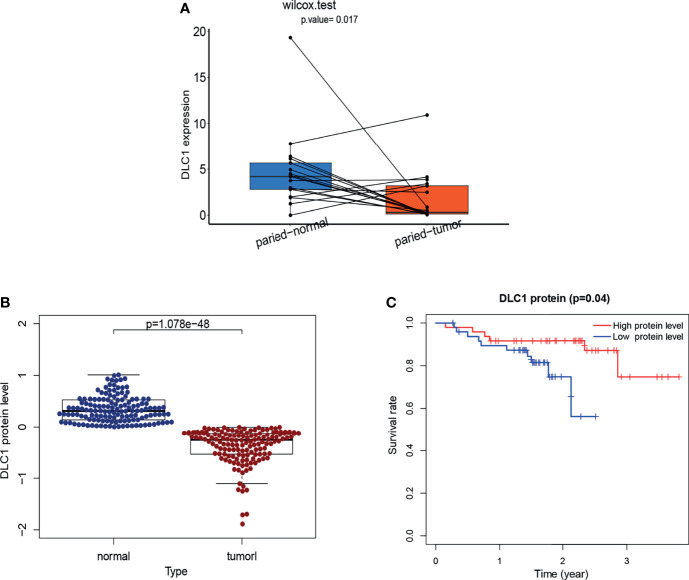
Validation of the target gene by RT-qPCR analysis and validation of DLC1 protein expression based on a dataset from cBioPortal. **(A)**Tumor tissues and paired normal tissues were derived from 17 patients with EC, and Wilcoxon signed-rank test was used for evaluating the statistical significance of differences. **(B)** Relationship between DLC1 protein expression and prognosis of EC in the cBioPortal database. Patients were divided into high-target protein expression or low-target protein expression group depending on the comparison with the median expression level using the log-rank test. **(C)** DLC1 protein expression in the normal and tumor samples. Analyses were performed across all normal and tumor samples with *P*-value close to zero using the Wilcoxon rank-sum test.

As shown in **Figure 4**, we found DLC1 with lower expression levels in tumor samples (**Figures 4A, B**); patients with the high expression of DLC1 exhibited prolonged survival (**Figures 2J–L**). We then collected 17 UCEC samples and adjacent normal tissue samples to validate the expression of DLC1 in patients with UCEC. The expression of DLC1 was consistently downregulated in tumor samples (**Figure 8A**).

### Target Protein Expression Validation

We download a dataset [Endometrial Cancer (MSK, 2012)] of 95 patients with UCEC (83 endometrioid and 12 serous tumors) from the cBioPortal database to investigate the endogenous protein levels of DLC1. The target protein level was consistently downregulated in tumor samples. Survival analysis demonstrated that the decrease in DLC1 protein level was related to a worse prognosis (*P* = 0.04) (**Figure 8B, C**).

## Discussion

This study sought to find out the correlation between DLC1 expression and EC prognosis through TCGA database and subsequently explore its related mechanism. DLC1 is associated with immune activity. Importantly, a series of bioinformatics analyses suggested that DLC1 might be an essential regulator of the TME status for patients with EC.

In this study, we assessed the expression of DLC1 in 33 different types of cancers using the TCGA database, so as to reveal the differences between tumor and normal tissue expression of DLC1 in many cancers. We explored the prognostic relation between the expression of DLC1 and 33 different types of cancers, indicating the correlation between lower DLC1 expression and a poor UCEC prognosis. DLC1 is viewed as a potential tumor suppressor. A study showed that restoring DLC1 expression in cancer cells induced apoptosis and senescence, inhibited migration and invasiveness, and reduced tumor formation (16). Suppressing DLC1 degradation could inhibit the migration through the DLC1/RhoA pathway in breast cancer (17). The upregulated expression of DLC1 could inhibit the migration from 32.5% to 11.5% in HCC cells, and its post-translational modification was mediated by PI3K/Akt signaling (18). The restoration of DLC1 expression in gallbladder cancer cells resulted in caspase-3‐mediated apoptosis (19). DLC1 may mediate cell apoptosis and suppress the growth and invasion through regulating the Wnt/β-catenin signaling pathway (20). Despite a series of studies focusing on DCL1, the mechanism of its influence on cancer cells has not yet been completely elucidated.

We further explored whether the expression of DLC1 was related to histological type, histological grade, clinical stage, BMI, diabetes, event, and menopause status. DLC1 expression was significantly lower in the late stages than in the early stages of cancer. These results suggested that DLC1 might serve as a prognostic marker of EC. A report showed that DLC1 was often lost in cancer cells. It remained within the stromal components and was concentrated in proximity to endothelial (CD34 positive) cells (21). Therefore, we further analyzed the relationship between DLC1 expression and TME. The GSEA results showed that the metabolic pathways including pyrimidine metabolism, pyruvate metabolism, alanine aspartate and glutamate metabolism, and oxidative phosphorylation were enriched in the low-DLC1 expression group. A study suggested that stroma-associated pancreatic stellate cells supported malignant cells by providing nutrients such as alanine and lipids to the TME (22). This might be part of the mechanisms underlying the relationship between DLC1 and tumor prognosis. In the high-DLC1 expression group, tumor immunity-related signaling pathways, such as Wnt signaling pathway, leukocyte transendothelial migration, and JAK–STAT signaling pathway, were markedly enriched. Our study found that the balance between typical tumor pathways and metabolism affected the immunity status to a certain degree. Accordingly, the downregulation of DLC1 with the advancing stage of UCEC and the conversion of TME from immune-predominant into metabolic-dominant status indicated that DLC1 might be a potential tumor suppressor in UCEC.

The CIBERSORT analysis for the proportion of TICs revealed that the number of macrophages M2 negatively correlated with DLC1 expression in patients with UCEC. Different TAM subsets play a role in the inhibition of tumor progression. The levels of anti-inflammatory cytokines, scavenging receptors, angiogenesis factors, and protease were higher in M2-like macrophages than in M1-like macrophages. Therefore, TAMs were deemed to promote tumor progression. Cytokines are highly dynamic in the TAM compartment. The macrophage-centric treatments include transformation into M1-like macrophages. A recent study demonstrated a cross talk between tumor cells and TAMs *via* SIGLEC1, CCL8, and CSF1 (13). We concluded that the expression of DLC1 negatively correlated with SIGLEC1 and CCL8 expression using the TIMER website, which further proved that DLC1 expression was negatively related to M2-like macrophages (**Supplement Table 3**). Therefore, our findings might offer new ideas for macrophage-related treatments in EC.

TAMs can be polarized into M2-like or M1-like macrophages (distinct TAM subsets) that can induce or repress antitumor immunity, angiogenesis, and cell migration (23). M2-like macrophages express higher levels of anti-inflammatory cytokines, scavenging receptors, angiogenesis factors, and proteases compared with M1-type macrophages (23, 24). In consequence, TAMs are confirmed to stimulate tumor progression. Cytokines within the TME compartment, which is highly dynamic, can manipulate immune functions (6, 25). TAM-centered therapeutic approaches include inducing TAMs to form M1-like macrophages (26).

Studies showed that a multidisciplinary approach to the study of tumors could better identify novel diagnostic and prognostic biomarkers based on the analysis of high-quality biobanks, as well as help formulate better management (27–30). In the final part of the study, we applied real-time quantitative PCR to determine the expression of DLC1 in EC and adjacent normal tissue samples. Then, the DLC1 protein level was assessed using a dataset from the cBioPortal database for final verification. Therefore, DLC1 is a valuable prognostic biomarker correlated with TME remodeling in EC. Finding an efficient method to upregulate DLC1 expression may provide an adjuvant treatment for patients with UCEC who are deficient in DLC1 expression.

## Conclusions

Our study first analyzed the expression of DLC1 in multiple tumor and normal tissue types using the TCGA database, displaying that DLC1 expression significantly declined in UCEC. We concentrated on DLC1 in UCEC for a subsequent set of analyses. We discovered that the decreased expression of DLC1 in EC predicted a poorer prognosis. GSEA was performed to further investigate the mechanism. The tumor immunity- and inflammation-related activities were mainly enriched in the high-DLC1 expression group, while metabolic pathways were enriched in the low-DLC1 expression group. Therefore, we postulated that DLC1 might be associated with the immune and stromal components in the TME. We applied for the CIBERSORT algorithm and discovered that resting memory CD4 T cells and resting mast cells positively correlated with DLC1 expression, but the number of macrophages M2 had a negative correlation. Finally, we applied real-time quantitative PCR to determine the expression of DLC1 in EC and adjacent normal tissue samples for further verification. Then, the DLC1 protein level was assessed using a dataset from the cBioPortal database for final verification. These results allowed a more comprehensive understanding of the mechanism of DLC1 affecting the EC prognosis, shedding a different light on the immune therapeutics of UCEC. However, the study had certain limitations in terms of the small number of human samples and the lack of functional studies on EC. More human samples and the corresponding clinical data should be collected to confirm the findings based on the databases.

## Data Availability Statement

The datasets presented in this study can be found in online repositories. The names of the repository/repositories and accession number(s) can be found in the article/**Supplementary Material**.

## Ethics Statement

The studies involving human participants were reviewed and approved by the Medical Ethics Committee of The Affiliated First Hospital of Fujian Medical University. The patients/participants provided their written informed consent to participate in this study.

## Author Contributions

YH and YW conceived of the presented idea. YW developed the theory and performed the computations. YW and SC collected samples. CL performed the real-time quantitative PCR. YW verified the analytical methods. LZ encouraged SC to investigate and supervise the findings of this work. YW and LZ wrote the manuscript with input from all authors. All authors contributed to the design and implementation of the research, to the analysis of the results, and to the writing of the manuscript.

## Conflict of Interest

The authors declare that the research was conducted in the absence of any commercial or financial relationships that could be construed as a potential conflict of interest.

## Publisher’s Note

All claims expressed in this article are solely those of the authors and do not necessarily represent those of their affiliated organizations, or those of the publisher, the editors and the reviewers. Any product that may be evaluated in this article, or claim that may be made by its manufacturer, is not guaranteed or endorsed by the publisher.
