# Supplementary material for: DLC1 Is a Prognosis-Related Biomarker Correlated With Tumor Microenvironment Remodeling in Endometrial Carcinoma
Source: Front Oncol. 2022 Feb 11;12:823018. doi: 10.3389/fonc.2022.823018 (PMC8874285; doi:10.3389/fonc.2022.823018)
Supplement: Supplementary file 3 [file Table_1.docx]

Supplement Table 1. Enriched gene sets.

| **MSigDB collection** | **Gene set name** | **NES** | **NOM *p*-val** | **FDR q-val** |
| --- | --- | --- | --- | --- |
| C2.cp.kegg.v7.2.symbols.gmt  DLC1 high expression    DLC1 low expression  c7.all.v7.2.symbols.gmt  DLC1 high expression | KEGG_ADHERENS_JUNCTION  KEGG_JAK_STAT_SIGNALING_PATHWAY  KEGG_LEUKOCYTE_TRANSENDOTHELIAL_MIGRATION  KEGG_MAPK_SIGNALING_PATHWAY  KEGG_PATHWAYS_IN_CANCER  KEGG_PROSTATE_CANCER  KEGG_TGF_BETA_SIGNALING_PATHWAY  KEGG_WNT_SIGNALING_PATHWAY  KEGG_ALANINE_ASPARTATE_AND_GLUTAMATE_METABOLISM  KEGG_DNA_REPLICATION  KEGG_OXIDATIVE_PHOSPHORYLATION  KEGG_PROTEASOME  KEGG_PYRIMIDINE_METABOLISM  KEGG_PYRUVATE_METABOLISM  KEGG_RNA_POLYMERASE  KEGG_SPLICEOSOME  GSE12366_GC_VS_MEMORY_BCELL_DN  GSE16385_IFNG_TNF_VS_UNSTIM_MACROPHAGE_ROSIGLITAZONE_TREATED_DN  GSE17974_IL4_AND_ANTI_IL12_VS_UNTREATED_12H_ACT_CD4_TCELL_UP  GSE17974_IL4_AND_ANTI_IL12_VS_UNTREATED_48H_ACT_CD4_TCELL_UP GSE21670_STAT3_KO_VS_WT_CD4_TCELL_IL6_TREATED_DN GSE21774_CD56_BRIGHT_VS_DIM_CD62L_POSITIVE_NK_CELL_UP  GSE25088_CTRL_VS_IL4_STIM_MACROPHAGE_UP GSE30962_ACUTE_VS_CHRONIC_LCMV_SECONDARY_INF_CD8_TCELL_UP GSE30971_CTRL_VS_LPS_STIM_MACROPHAGE_WBP7_KO_4H_UP GSE37301_HEMATOPOIETIC_STEM_CELL_VS_RAG2_KO_NK_CELL_DN GSE39556_UNTREATED_VS_3H_POLYIC_INJ_MOUSE_NK_CELL_DN GSE39820_TGFBETA1_IL6_VS_TGFBETA1_IL6_IL23A_TREATED_CD4_TCELL_UP GSE40274_CTRL_VS_FOXP3_TRANSDUCED_ACTIVATED_CD4_TCELL_DN GSE4590_LARGE_PRE_BCELL_VS_VPREB_POS_LARGE_PRE_BCELL_DN | 1.831  1.801  1.864  2.026  1.826  1.891  2.227  2.048  -1.725  -1.823  -2.218  -2.015  -1.766  -1.862  -2.061  -1.790  2.310  2.236  2.368  2.310  2.385  2.314  2.345  2.247  2.153  1.749  2.466  2.252  2.241  2.237 | 0.017  0.010  0.004  0  0.008  0.006  0.002  0  0.026  0.016  0  0.002  0.012  0.014  0  0.033  0  0  0  0  0  0  0  0  0  0.008  0  0  0  0 | 0.044  0.044  0.035  0.014  0.039  0.036  0.002  0.018  0.066  0.040  0.003  0.013  0.053  0.050  0.012  0.048  0.004  0.006  0.002  0.004  0.002  0.004  0.003  0.005  0.007  0.030  0.001  0.006  0.006  0.006 |

NES: normalized enrichment score; NOM: nominal p-value; FDR: false discovery rate. Gene sets with NOM p-value less than 0.05 and FDR q-value less than 0.07 were considered as statistical significance. Only several leading sets enriched in DLC1 high expression both in KEGG and C7 were listed here due to the large number of enriched gene sets.
