# Supplementary material for: DLC1 Is a Prognosis-Related Biomarker Correlated With Tumor Microenvironment Remodeling in Endometrial Carcinoma
Source: Front Oncol. 2022 Feb 11;12:823018. doi: 10.3389/fonc.2022.823018 (PMC8874285; doi:10.3389/fonc.2022.823018)
Supplement: Supplementary file 4 [file Table_2.docx]

Supplement Table 2. TICs co-determined by difference test and correlation test

| TICs | Correlation test（*p*-value） | | Difference test（*p*-value） |
| --- | --- | --- | --- |
| T cells CD4 memory resting  Mast cells resting  Macrophages M2 | 0.260(<0.001)  0.160 ( 0.013)  -0.190 (0.002) | 0.010  0.040  0.005 | |
