# Supplementary material for: DLC1 Is a Prognosis-Related Biomarker Correlated With Tumor Microenvironment Remodeling in Endometrial Carcinoma
Source: Front Oncol. 2022 Feb 11;12:823018. doi: 10.3389/fonc.2022.823018 (PMC8874285; doi:10.3389/fonc.2022.823018)
Supplement: Supplementary file 5 [file Table_3.docx]

**Supplement Table 3: Correlation analysis between DLC1 and relate genes and markers of immune cells in TIMER.**

| **Description** | **Gene markers** | **UCEC** | | | |
| --- | --- | --- | --- | --- | --- |
|  | | **None** | | **Purity** | |
|  |  | **Cor** | **P** | **Cor** | **P** |
| TAM | SIGLEC1 | -0.207 | *** | -0.27 | *** |
|  | CCL8 | -0.137 | * | -0.228 | *** |
|  | CSF1 | 0.127 | * | 0.056 | 0.34 |
|  | IL10 | 0.210 | *** | 0.168 | * |
| M2 Macrophage | CD163 | 0.096 | 0.024 | 0.029 | 0.627 |
|  | VSIG4 | 0.102 | 0.017 | 0.001 | 0.986 |
|  | MS4A4A | 0.209 | *** | 0.137 | 0.019 |
