# Supplementary material for: DLC1 Is a Prognosis-Related Biomarker Correlated With Tumor Microenvironment Remodeling in Endometrial Carcinoma
Source: Front Oncol. 2022 Feb 11;12:823018. doi: 10.3389/fonc.2022.823018 (PMC8874285; doi:10.3389/fonc.2022.823018)
Supplement: Supplementary file 6 [file Table_4.docx]

**Supplement Table 4. The association between DLC1 expression and cancer patient prognosis.**

| **Cancer** | **OS** | | | **DSS** | | | **PFI** | | |
| --- | --- | --- | --- | --- | --- | --- | --- | --- | --- |
|  | **HR** | **95%CI** | **P** | **HR** | **95%CI** | **P** | **HR** | **95%CI** | **P** |
| ACC | 1.777 | 1.166-2.708 | **0.007** | 2.017 | 1.306-3.115 | **0.002** | 2.156 | 1.544-3.011 | **<0.001** |
| LGG | 2.431 | 1.875-3.152 | **<0.001** | 2.765 | 2.091-3.655 | **<0.001** | 2.178 | 1.740-2.725 | **<0.001** |
| KIRC | 0.682 | 0.564-0.826 | **<0.001** | 0.618 | 0.491-0.777 | **<0.001** | 0.738 | 0.612-0.890 | **0.001** |
| UVM | 0.287 | 0.154-0.537 | **<0.001** | 0.259, | 0.131-0.513 | **<0.001** | 0.417 | 0.244-0.713 | **0.001** |
| UCEC | 0.556 | 0.383-0.806 | **0.002** | 0.544 | 0.353-0.837 | **0.006** | 0.676 | 0.509-0.898 | **0.007** |

Bold values indicate P<0.05.HR, hazard ratio; CL, confidence interval.
